# A Treatise on Diseases of the Skin

**Published:** 1906-04

**Authors:** 


**A Treatise on Diseases of the Skin**. For the use of advanced Students and Practitioners. By **Henry W. Stelwagon**, M.D., Ph. D., Professor of Dermatology, Jefferson Medical College, Philadelphia; and Clinical Professor of Dermatology, Woman's Medical College, Philadelphiia. Fourth Edition, revised. Octavo, 1135 pages, 258 text-illustrations, 32 full page lithographic and half-tone plates. Philadelphia and London: W. B. Saunders & Co. 1905. (Price, Cloth, $6.00, Sheep or Half-morocco, $7.00, net).

The fact that this admirable work has reached its fourth edition in so short a period—namely, since 1901 is alone sufficient to indicate its superior value, especially when we take into consideration the many other good works which have preceeded it. The author follows the logical plan for such a work, and the treatment of his subject is fresh and up-to-date, including the now-conceded value of the *x*-ray in connection with therapeutics, as well as the ultra violet, Finsen and other modern treatments. Throughout the book all important references are given, and upon many of the pages a bibliography is found which is of great value. The work includes all the more recent interpretations of the diseases of which it deals.

Throughout the entire volume Stelwagon shows the results of his fruitful experience as a teacher, by the interpretation of a subject which is not easily dealt with. This he has done well, and in a concise and comprehensive manner that, in itself, constitutes a recommendation. Certainly it is no trifle to treat a subject of this kind in such a manner that it will leave a definite and permanent impression upon the mind of the student.

One of the most noteworthy changes is the suppression of Mracek colored pictures and the substitution of a few of the author's own. These are, perhaps, colored too highly, but they are an improvement upon the old ones. The numerous black and white illustrations are exceptionally good, being original, new and striking. For our part we say always omit colors when the details of the disease can be so well maintained as they are in Stelwagon's photographic illustrations.

The book is splendidly written, with a masterly discussion of the principles of dermatology. It is a pleasure to note the advance in dermatological methods in these days of specialists as here so pointedly illustrated.

